# Unraveling Origin, History, Genetics, and Strategies for Accelerated Domestication and Diversification of Food Legumes

**DOI:** 10.3389/fgene.2022.932430

**Published:** 2022-07-22

**Authors:** Ambika, Muraleedhar S. Aski, Gayacharan, Aladdin Hamwieh, Akshay Talukdar, Santosh Kumar Gupta, Brij Bihari Sharma, Rekha Joshi, H. D. Upadhyaya, Kuldeep Singh, Rajendra Kumar

**Affiliations:** ^1^ Department of Genetics and Plant Breeding, University of Agricultural Sciences, GKVK, Bangalore, India; ^2^ Division of Genetics, Indian Agricultural Research Institute (ICAR), New Delhi, India; ^3^ National Bureau of Plant Genetic Resources (ICAR), New Delhi, India; ^4^ International Center for Agriculture Research in the Dry Areas (ICARDA), Giza, Egypt; ^5^ National Institute of Plant Genome Research (NIPGR), New Delhi, India; ^6^ International Crops Research Institute for the Semi-Arid Tropics (ICRISAT), Patancheru, India; ^7^ University of Georgia, Athens, GA, United States

**Keywords:** evolution, divergence, diversification, domestication, domestication syndrome, pulse crop

## Abstract

Domestication is a dynamic and ongoing process of transforming wild species into cultivated species by selecting desirable agricultural plant features to meet human needs such as taste, yield, storage, and cultivation practices. Human plant domestication began in the Fertile Crescent around 12,000 years ago and spread throughout the world, including China, Mesoamerica, the Andes and Near Oceania, Sub-Saharan Africa, and eastern North America. Indus valley civilizations have played a great role in the domestication of grain legumes. Crops, such as pigeon pea, black gram, green gram, lablab bean, moth bean, and horse gram, originated in the Indian subcontinent, and Neolithic archaeological records indicate that these crops were first domesticated by early civilizations in the region. The domestication and evolution of wild ancestors into today’s elite cultivars are important contributors to global food supply and agricultural crop improvement. In addition, food legumes contribute to food security by protecting human health and minimize climate change impacts. During the domestication process, legume crop species have undergone a severe genetic diversity loss, and only a very narrow range of variability is retained in the cultivars. Further reduction in genetic diversity occurred during seed dispersal and movement across the continents. In general, only a few traits, such as shattering resistance, seed dormancy loss, stem growth behavior, flowering–maturity period, and yield traits, have prominence in the domestication process across the species. Thus, identification and knowledge of domestication responsive loci were often useful in accelerating new species’ domestication. The genes and metabolic pathways responsible for the significant alterations that occurred as an outcome of domestication might aid in the quick domestication of novel crops. Further, recent advances in “omics” sciences, gene-editing technologies, and functional analysis will accelerate the domestication and crop improvement of new crop species without losing much genetic diversity. In this review, we have discussed about the origin, center of diversity, and seed movement of major food legumes, which will be useful in the exploration and utilization of genetic diversity in crop improvement. Further, we have discussed about the major genes/QTLs associated with the domestication syndrome in pulse crops and the future strategies to improve the food legume crops.

## Introduction

Food legumes are a key component of the agricultural ecosystem. These plants are a chief member of the most diverse and ecologically crucial botanical families (Lewis, 2005; Legume Phylogeny Working Group (LPWG, 2017; Zhao et al., 2021). Worldwide, food legumes are grown in 93.18 Mha with an annual production volume of 89.82 million tons and a productivity average of 963.9 kg/ha (FAOSTAT, 2020). Legumes play a vital role in crop rotations or intercropping schemes as these plants are capable of nitrogen assimilation through a symbiotic relationship with rhizobia. The use of food legumes in crop rotations has dropped since the Green Revolution, as has their consumption, resulting in nutritional imbalances, such as protein and vitamin deficiency, as well as an excessive reliance on nitrogenous fertilizers in agricultural systems, which causes environmental pollution (Foyer et al., 2016; Considine et al., 2017; Adams et al., 2018). Legume crops play a significant role in increasing indigenous nitrogen production in addition to meeting demands of human population for protein and energy. Leguminous crop farming in rotation with nonleguminous crops improves soil fertility by restoring natural soil matter and limiting pest-related diseases. Legumes are very rich in protein consequently have abundant nitrogen content. Most of the crop plants incorporate carbon in the environment in contrast to nitrogen; nevertheless, the important microorganisms necessary for healthy soil need both carbon and nitrogen. The natural soil development process needs nitrogen and carbon elements as its constituents, which provide conducive ambient for action of microorganisms for the decomposition of crop residues. The leguminous plants encourage earthworm growth in the soil which facilitate the organic matter decomposition, enhance nutrient availability to plants and improve soil structure loosening the soil, soil aeration and root growth and development. The improved soil structure enhances air flow and water movement in the soil. The deep root system provided by legumes aids in recycling the crop nutrients in a deep soil profile. These measures collectively promote ecologically effective utilization of fertilizers and avert nutrient loss, especially nitrate–nitrogen, due to leaching into the soil. An important protein of nitrogen fixing symbiosis, glomalin, assists in binding the soil to form stable composite matter. This stability of the soil strengthens the soil structure through pore space and tilth, thus preventing soil erosion and crusting. Legumes also aid in reducing soil pH and provide a favorable environment for a constructive plant–soil–microbe interaction for optimum crop growth and development.

Food legumes help in providing food security by safeguarding the human health, reducing climate change, and boosting biodiversity. Legume crops are important sources of human nutrition, animal feed, and raw products. Legumes provide great nutritional value through the improvement of dietary fiber, vitamins, and minerals and are one of the most important plant-based protein sources in human diets (Mudryj et al., 2014). Leguminous plants are an exorbitant source of protein diet, which are rich in essential amino acid lysine (20–45% of total protein) (Philips, 1993). Cereals are high in sulfur-containing amino acids, and legumes are high in lysine amino acids. These two crops complement each other in nutrient value (Staniak et al., 2014). Thus, legumes and cereals together as a diet significantly improve the protein uptake of the population. The cultivation of legumes will positively result in the production of dietary food, which will certainly improve the affordability of food items to low-income groups to ease malnourishment. In developing countries, malnourishment in lactating women and children, due to lack of protein rich diet is a major issue (Staniak et al., 2014). Malnourishment is prevalent in lower income group in developing countries because of unaffordability of regular supply of animal protein sources such as egg, meat, and milk. As a result, the FAO emphasized and stated that adding legumes to the human diet can help in the fight against nutritional challenges, such as malnutrition, micronutrient deficiencies, obesity, and food-related diseases, all of which are common in many countries.

Around 10,000 years ago, the first crops were domesticated in the Fertile Crescent, and agriculture began (Brown et al., 2009). Domestication is a dynamic and continuous process of converting wild species into cultivated species by selecting desirable characteristics in agricultural plants to meet human demands (Acquaah, 2009). Cultivators sought plants that have been domesticated for various desired attributes in diverse agroecological locations around the world. Domestication is the artificial selection of crop plants with desired qualities such as taste, yield, storage, and cultivation practices (Begna, 2020). Plant domestication by humans began around 12,000 years ago in the Fertile Crescent. It expanded throughout the world, including China, Mesoamerica, the Andes, and Near Oceania (all 10,000 years ago), Sub-Saharan Africa (8,000 years ago), and eastern North America (6,000 years ago) (Meyer et al., 2012). Crop plants have evolved as human behavioral ecology shifted from hunting and gathering to farming to meet our food requirements (Roth, 2006). During the transition phase of human behavior change and the inception of agriculture, women played a major role in seed selection and crop domestication (Roth, 2006).

Domesticated plant species are found in over 160 taxonomic groupings (Meyer et al., 2012). The most important families are Poaceae, Fabaceae, and Brassicaceae. Approximately 2,500 species have been partially domesticated (Dirzo and Raven, 2003), but only 250 are fully trained (Duarte et al., 2007; Harlan et al., 2012). Many of these are only used in specific situations or locations.

## Evolution and Divergence of Food Legumes

### Origin of Food Legumes

**FIGURE 1:**
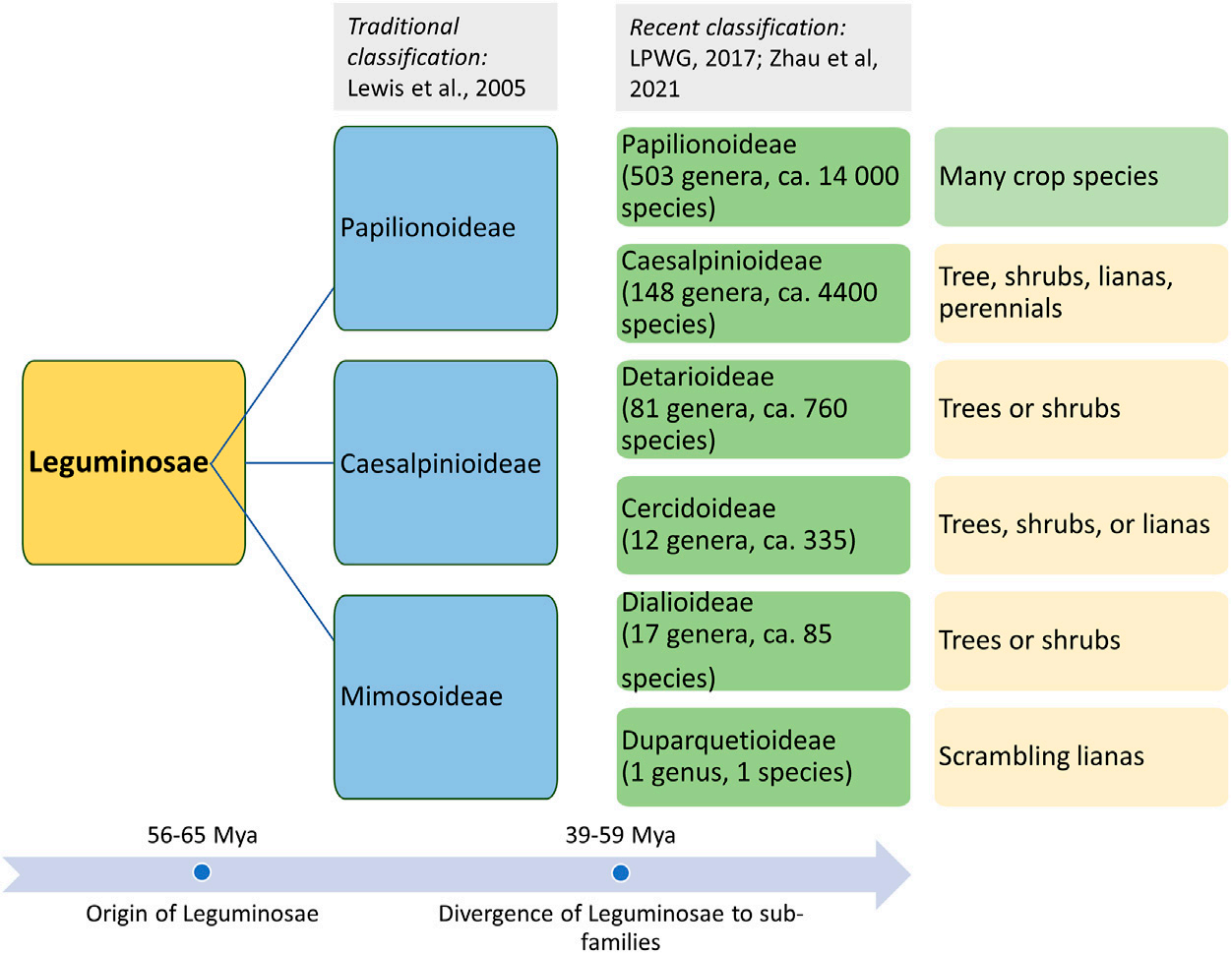
Origin of the Leguminosae family and classification of its members in three major subfamilies (traditional classification) and six subfamilies based on recent phylogenetic studies.

Life in the form of land plants evolved during the Paleozoic era (ca. 470 Mya). Several morphotypes evolved through the complex processes of genetic mechanisms and selection for evolved morphotypes, which led to the evolution of different plant species. However, their evolutionary history is not well established because of the very sparse information on fossil records for plant progenitors and morphotypes. This has led to alternative interpretations of species lineages based on the radiation of plant forms by comparing the living descendants (phylogenetic approaches). The evolution of flowering plants (angiosperms) is accepted to have occurred during the early Cretaceous epoch (145–100.5 Mya). However, a recent finding on angiosperm-like pollen from Northern Switzerland dates back to the Middle Triassic period (247.2–242.0 Mya) (Hochuli and Feist-Burkhardt, 2013). The divergence and evolution of Leguminosae based on the fossils and phylogenetic records of plants are also speculative (Pratap and Kumar, 2011). There are several reports of fossils similar to a legume-like structure, but they could not be assigned unequivocally as legumes’ fossils. The first definitive fossil record of legumes dates back to the Late Paleocene (ca. 56 Mya) (Herendeen, 2001). Fossil records and phylogenetic studies indicate that the members of the legume family originally evolved during the early Tertiary period in arid and semiarid regions along the Tethys seaway (Herendeen, 1992). Although legumes’ diversity is highly documented in tropical and subtropical regions, fossil records contradict this theory of Mesozoic origin and diversification. Another speculation is the “moist equatorial megathermal” origin of legumes during the mid to early Cretaceous period (Mesozoic era), which also supports the West Gondwanan hypothesis for the legume origin (Morley, 2000). The understanding of the origin of Leguminosae plants based on fossil records and phylogenetics has become further more complex because of the mass extinction that happened during the Cretaceous–Paleogene boundary (KPB; 65 Mya) (Koenen et al., 2021). KPB is not considered the major mass extinction event for plants; rather, it led to the sudden increase in plant origination and diversification (Silvestro et al., 2015). A similar rise in plant origination and diversification was observed during the global aridification in the Miocene (ca. 10–5 Mya). Fossil records suggest that legumes were ecologically (co-)dominant across the various types of vegetation and the probable reason for their prominence was KPB-driven favorable conditions and frequent whole genome duplication (WGD) (Koenen et al., 2021). On the basis of the molecular studies, molecular clock estimation also revealed an early radiation of subfamilies near the KPB mass extinction, followed by a major divergence event that happened within ca. 15 million years (Zhao et al., 2021). WGD events across legumes and allopolyploidy events among the earliest lineages in the early phase of legume evolution are considered the major factors for legume evolution and divergence into various clades and subclades. At present, over 19,000 extant species of the Leguminosae family are present in almost every kind of ecological settings (Lewis et al., 2005). The classification of the Leguminosae family is given in Figure 1.

### Divergence of Food Legumes

**FIGURE 2:**
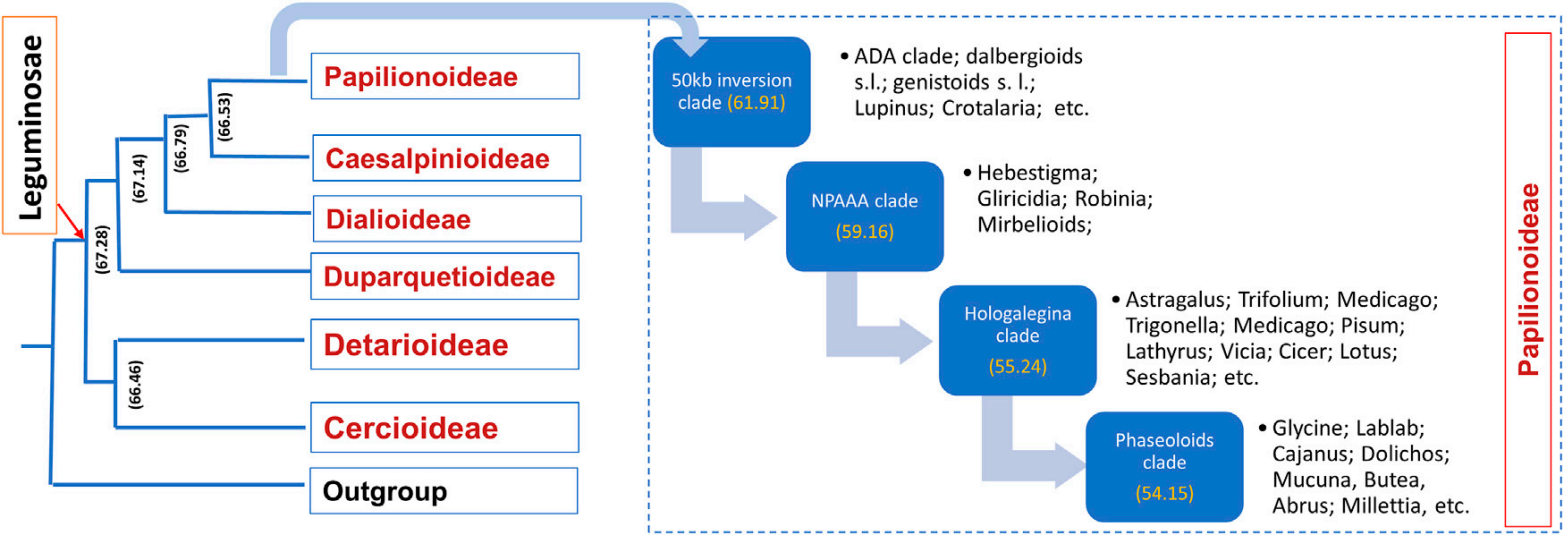
Time divergence of major legume clades. Values inside the brackets are the estimated time of divergence based on phylogenomic studies (source: Zhao et al., 2021).

Leguminosae is the second largest family of angiosperms after the Poaceae in terms of their agricultural importance. Overall, Leguminosae is the fourth largest family on Earth after Asteraceae and Orchidaceae. The Leguminosae family consists of 765 genera and 19,500 species (LPWG, 2017; Zhao et al., 2021). Leguminosae as a monophyletic origin is strongly supported by all molecular studies. On the basis of the flower structure, the Leguminosae was divided into three subfamilies traditionally, namely, Caesalpinioideae, Mimosoideae, and Papilionoideae (Lewis et al., 2005). Recent studies based on extensive molecular data on chloroplast sequence, plastome, or nuclear genes all support the new classification that contains six subfamilies, viz., Papilionoideae, Caesalpinioideae, Detarioideae, Cercidoideae, Dialioideae, and Duparquetioideae (LPWG, 2017; Zhang R. et al., 2020; Koenen et al., 2020). Among these six subfamilies, Detarioideae, Caesalpinioideae, and Papilionoideae are further divided into tribes containing one or more genera. However, many of the tribes and genera are not monophyletic, and in several instances, the relationship among tribes and genera remains unclear (LPWG, 2017). Such classification ambiguities are more in the largest subfamily, Papilionoideae. However, recent advances in sciences and molecular tools are now making it is easier to understand evolutionary events, divergence, and interrelationships at various taxa levels. An extensive study was done to establish a robust phylogenetic relationship among the members of the Leguminosae family using 463 legumes belonging to 333 genera from six subfamilies, including other eudicot species (Zhao et al., 2021). In this study, phylogenomics and transcriptomics data on thousands of gene families revealed 28 putative whole genome duplication/triplication in Leguminosae, including the ancestors of Leguminosae. The divergence time of major Leguminosae (Fabaceae) clades is given in Figure 2. The subfamily Papilionoideae contains the highest number of legume crops that are in cultivation. The importance of this family to humankind is evidenced from their role in agriculture since its origin. Among the eight founder crops of agriculture, four are legumes, viz., lentil, pea, chickpea, and vetches. Legumes remain the second most important crop group after cereals.

## History of Domestication of Food Legumes

When agriculture originated in the Near East, food legumes were planted as companion crops to wheat and barley (Kislev and Bar-Yosef, 1988; Zohary and Hopf, 1973), whereas other key grain legumes had their domestication roots in Asia and the New World (Kislev and Bar-Yosef, 1988). Out of eight founder crops since the origin of agriculture, four were food legume crops, viz., lentil (*Lens culinaris* L.), pea (*Pisum sativum* L.), chickpea (*Cicer arietinum* L.), and bitter vetch (*Vicia ervilia*). According to Bahl et al. (1993), lentil was possibly the first grain legume to be domesticated in 11,000 BC. Along with other early domestications [such as *Phaseolus vulgaris* (L.) and *Glycine max* (L.) Merr.], pulses have continually been domesticated as agriculture has expanded and intensified, with more recent domestications, such as pigeon pea [*Cajanus cajan* (L.) Millsp.] and mung bean [*Vigna radiata* (L.) R. Wilczek], around 4,000 years ago in South Asia (Fuller and Harvey, 2006; Krieg et al., 2017), alfalfa (*Medicago sativa* L.) domesticated in Roman times (Prosperi et al., 2014), and narrow-leaved lupine (*Lupinus angustifolius* L.) domesticated as a sweet lupine over the past century (Gladstones, 1970). For a few food legumes, such as faba bean (*Vicia faba* L.), the nature of domestication has been obscured by the absence of a known compatible wild relative, although archaeological evidence is starting to clarify at the least the chronology of domestication (Caracuta et al., 2017). In India, food legumes, including green gram and black gram, were grown at various Harappan sites and at Balathal in Rajasthan. Horse gram was domesticated in South India, and it is a known form of Late Harappan Hulas.

### Chickpea (*Cicer arietinum* L.)

Chickpea is one of the eight “founder crops” that gave rise to agriculture. Chickpea was domesticated in the Fertile Crescent 12,000–10,000 years ago (Kislev and Bar-Yosef, 1988). The earliest records of chickpea used as food was in the 8th millennium BC at Tell el-Kerkh and Tell Abu Hureyra, Syria. However, in Tell el-Kerkh, both *Cicer arietinum* and the progenitor *Cicer reticulatum* Ladiz. were clearly identified, this being the earliest date for the cultivation of chickpea. It is worth noting that, unlike cultivated species, the wild progenitor’s origin is limited to a particular geographic area. Domesticated chickpeas have been discovered in archaeological sites dating back to the Pre-Pottery Neolithic period, which suggests that chickpea was confined to Fertile Crescent only until the early Neolithic era. However, in the late Neolithic era, chickpea had spread to modern Greece (Redden and Berger, 2007). The presence of chickpea seeds in the Nile valley are available at least as far back as the New Kingdom (1580–1100 BC). The seeds are still conserved at Cairo Museum (personal communication). However, the much older archaeological records of domesticated chickpea are available as far back as 3,300 BC onward in Egypt and the Middle East. During the Bronze Age (from 3,300 BC to 1,200 BC), chickpea also reached Crete in the West and eastward to the Indian subcontinent. By the Iron age (1200 BC to 600 BC), chickpea cultivation spread to South and West Asia, the Nile valley, and Ethiopia (Redden and Berger, 2007). The Spanish and Portuguese introduced chickpea to the New World in the 16th century. The linguistic naming of large-seeded chickpea as Kabuli chana (chickpea) indicates that Kabuli-type chickpea reached India from the Mediterranean region through Afghanistan in the 18th century (van der Maesen, 1987). N.I. Vavilov designated two primary centers of chickpea diversity, i.e., Southwest Asia and the Mediterranean, and one secondary center of diversity, i.e., Ethiopia (van der Maesen, 1987).

Later in the domestication process, the chickpea seeds spread to other parts of the world and niche-specific diversity evolved. As a result, the cultivated chickpea has two distinct seed forms, viz., desi and Kabuli type (Warkentin et al., 2005). Desi type is small-seeded, angular-shaped, and colored seeds with a higher percentage of fiber. Kabuli type is large-seeded, owl-shaped, beige-colored seeds with a low rate of fiber. Of late, a third category has been added to classify the chickpea seed, i.e., intermediate type. The medium type of chickpea is pea-shaped, smooth, and round. On the basis of the current needs, the cultivated chickpea is still evolving and reshaping its genome primarily for its plant type, nutrition, and resistance to environmental stresses.

### *Vigna* spp.

The genus *Vigna* consists of a large group of cultivated and wild relatives distributed across Asia and Africa. It comprises around seven subgenera and 19 sections with around a hundred species, out of which 7 (She et al., 2015) or 10 (Takahashi et al., 2016) species are most commonly cultivated worldwide. Cowpea (*Vigna unguiculata*) is the major food legume crop of this genus in its production and area under cultivation, and it is grown in ∼12 million ha, making it the third most important legume crop in the world. The two species, viz., *Vigna unguiculata* L. and *Vigna subterranean* L., are of African origin. The other five species are from Asia, known to have originated in the Indian subcontinent (*V. radiata* L., *V. mungo* L., and *V. aconitifolia* Jacq.) and in far East Asia (*V. angularis* Willd. and *V. umbellate*) (Smartt, 1985). However, some studies indicate that *Vigna* might have first evolved in Africa (Vaillancourt et al., 1993; Thulin et al., 2004).

Green gram [*V. radiata* (L.) R. Wilczek] and black gram [*V. mungo* (L.) Hepper] have been domesticated in Southeast Asia (Chandel et al., 1984; Smýkal et al., 2015). The progenitor species of both crops are found widely distributed in the Western Ghats and adjoining areas of India (Bisht et al., 2005). *V. radiata* var*. sublobata* (Roxb) and *V. mungo* var. *silvestris* (Lukoki, Marechal, and Otoul) are the progenitor species of green gram and black gram, respectively (Chandel et al., 1984). Neolithic archaeological evidences from Indus valley civilizations consistently indicated the Neolithic domestication of green gram and horse gram in different parts of India (Fuller, 2011). On the basis of the archaeological evidences and ecological settings, mung bean probably was first domesticated in the sides of Southern Peninsula, more precisely, north of the Krishna River (Fuller, 2011). Evidences from east Harappan zones indicated that the small-seeded mung bean (before the seed size increases) was probably introduced to the Ganges region from the south (Fuller, 2011). Early finds of the black gram come from Gujarat and the Northern Peninsula in India, where its progenitor species abundantly exist (Fuller and Harvey, 2006). Archaeological shreds of evidence dating back to 3,500–3,000 BC and genetic diversity studies indicate that the mung bean was domesticated in India (Fuller, 2007; Smýkal et al., 2015). On the basis of the genome sequence comparison for divergence between *V. radiata* var. *radiata* and *V. radiata* var. *sublobata* and also between *V. radiata* var*. radiata* (VC 1973A) and *V. radiata* var*. radiata* (V2984), mung bean domestication is predated to 4,000–6,000 years ago (Kang et al., 2014). The domesticated mung bean has spread to Southeast Asia and East Asia from India through different routes (Keatinge et al., 2011). First, it might have probably reached China *via* the Silk Road, and subsequently, the mung bean had spread to Southeast Asia (Kang et al., 2014).

Moth bean (*V. aconitifolia* (Jacq.) Marechal) is an underutilized, minor grain legume. The progenitor species of moth bean is presumed as *Phaseolus trilobata* (L.) [syn. *Phaseolus trilobus*, *Vigna trilobata* (L.) Verdc.], which is an endemic species of India and is found in other adjoining areas. Some studies contradict *V. trilobata* as progenitor species of moth bean. Takahashi et al. (2016) have tried to differentiate between cultivated and wild forms of *V. aconitifolia* morphologically. However, the cultigens and wild conditions are not very distinguishable, unlike the other *Vigna* species such as green gram, black gram, and cowpea. Probably the species is still in the active domestication process and needs a lot of improvement in agronomical and yield traits. Moth bean is commonly cultivated in India’s northern and western parts, particularly in dry regions of Rajasthan, Gujarat, and Madhya Pradesh (Bisht et al., 2005). However, it is also found sporadically in Pakistan, Myanmar, and Sri Lanka (Jain and Mehra, 1980). On the basis of the prevalence of cultivated and wild forms, India is presumed to be the center of origin of the moth bean (Vavilov, 1926; de Candolle, 1985). Marechal et al. (1978) proposed Sri Lanka and Pakistan as the centers of diversity of moth bean. It is the most drought hardy and heat-tolerant food legume among Asian Vignas.

The cowpea (*Vigna unguiculata* L. Walp.) is the most important food legume crop in the genus *Vigna* in terms of production and area coverage, as well as the world’s third most important legume crop. Cowpea is an important crop in the semiarid and subhumid zones of Africa and Asia, where it is an important part of the Sub-Saharan African diet. It is tolerant of marginal and changing environments, making it one of Sub-Saharan Africa’s most important foods (Smýkal et al., 2015). Cowpea is thought to have originated in central–southern Africa, with West Africa and India as the first and second most likely domestication centers, respectively (Perrino et al., 1993; Singh, 1997). The first archaeological evidence of cowpea cultivation in Africa, dated from 1830 to 1595 BC, was identified by D'Andrea et al. (2007). *V. unguiculata* var. *spontanea* (previously var. *dekindtiana*) is the wild progenitor species of cowpea. It is widely dispersed across Africa (Padulosi and Ng, 1997). Cowpea domestication resulted in significant phenotypic modifications such as reduced pod shattering, increased grain size, and reduced flowering time. The genetic basis for these modifications is poorly understood. Cowpea domestication has resulted in a more consistent growth habit, larger pods and seeds, earlier flowering, and reduced pod cracking. Wild cowpeas feature purple flowers and dark mottled seed coats, whereas cultivated cowpeas have a wide range of flower and seed coat colors.

### Soybean (*Glycine max* L.)

Soybean (*Glycine max* L.) is primarily grown for its oil content in grains. It is one of the oldest crops of the world (Hymowitz, 1970). There are several archaeological, historical, and cultural evidences indicating the earliest known records of soybean cultivation in China, which proves China as the possible center of origin for soybean. The word “Shu,” which means soybean in Chinese language, is found written in many ancient books of China. An ancient inscription of a soybean word (ca. 3,700 years old, during the Yin and Shang dynasties) on bones and tortoise shell was found in China. In addition, the excavations in the 2,600 year old Dahaimeng site in Yongji County found carbonized soybean seeds. There are also other archaeological sites (3,000 years old), such as the Damudan Tun Village in Heilongjiang Province, where remains of soybean seed were found. The exact date of the commencement of soybean cultivation is unknown. However, early bronze inscriptions indicate that soybean cultivation may have begun during the Shang Dynasty (1500–1100 BC) (http://www.soymeal.org/FactSheets/HistorySoybeanUse.pdf). The oldest known evidence of human use of *Glycine* spp. comes from a Neolithic site in Jiahu, Henan Province, where charred soybean remnants were discovered.^1^ *Glycine soja* L. Merril., an endemic species of China, is considered the wild progenitor species of the cultivated soybean. Genome sequence information also indicates *G. soja* as the progenitor species of *G. max*, the cultivated species of soybean (Kim et al., 2010). The loss of pod dehiscence in *G. soja* was the major change, which leads to the domestication of the crop (Funatsuki et al., 2014).

### Pea (*Pisum sativum* L.)

Pea (*Pisum sativum* L.) was domesticated about 10,000 years ago in the Mediterranean region, particularly in the Middle East (Ambrose, 1995; Daniel and Maria, 2000). Peas are currently classified into three types: *Pisum sativum* L. grows from Iran and Turkmenistan to Asia, northern Africa, and southern Europe; *Pisum fulvum* (Sibth. and Smith.) grows in Syria, Lebanon, Jordan, and Israel; and *Pisum abyssinicum* (A. Braun) grows from Yemen to Ethiopia.^2^ Both *Pisum sativum* and *Pisum fulvum* were domesticated around 11,000 years ago in the Near East from an extinct parent of *Pisum* spp., and *P. abyssinicum* was developed independently of *P. sativum circa* 4,000–5,000 years ago in Old Kingdom or Middle Kingdom Egypt (Smýkal et al., 2011). *Vavilovia formosa* was added and classified in the tertiary gene pool (Smýkal et al., 2011). The phylogenetic status of the monotypic genus *Vavilovia* was studied using nrDNA ITS and cpDNAtrnL-F and trnS-G regions, and *Vavilovia* was found to be closely related to *Pisum*, forming a group that is close to *Lathyrus*. Molecular data and some morphological and biological characteristics strongly indicated that *Vavilovia* should be subsumed under *Pisum* as *Pisum formosum* (Oskoueiyan et al., 2010).

### Common Bean (*Phaseolus vulgaris* L.)

The common bean (*Phaseolus vulgaris* L.) was domesticated in Mesoamerica and the Andes mountains some 5,000 years ago. Numerous studies have been conducted to identify and trace the crop’s complicated evolutionary and domestication history (Bitocchi et al., 2012; Bellucci et al., 2014; Schmutz et al., 2014). Based on a genomic sequence variation at five loci on a large sample set representing the entire geographical distribution of wild-forms establishes the Mesoamerican origin of common bean (Bitocchi et al., 2012). The study also indicated a severe bottleneck preceded by the common bean domestication, and seed spread from Mesoamerica to the Andes mountains happened. A recent phylogenomics study based on sequencing of nuclear and chloroplast genomes of 29 accessions representing 12 *Phaseolus* species revealed a major speciation event in tropical Andes that gave rise to a sibling species, formerly considered the wild ancestor of *P. vulgaris* (Rendón-Anaya et al., 2017). This study revealed the divergence of the ancestor prior to the split of Mesoamerican and Andean common bean gene pools. Population structure study also revealed that the Andes and northern Peru–Ecuador gene pools from South America originated from two separate migration events from Mesoamerica (Bitocchi et al., 2012). *P. vulgaris* is perhaps the most economically important species in the genus *Phaseolus*. *P. lunatus* L. (lima bean), *P. coccineus* L. (runner bean), *P. dumosus* Macfad. (year-long bean), and *P. acutifolius* A. Gray are some of the other domesticated common bean species (tepary bean) under cultivation. *P. vulgaris* and *P. lunatus* are found wild in Mesoamerica and South America, respectively, but *P. dumosus*, *P. coccineus*, and *P. acutifolius* are only found in Mesoamerica (Bitocchi et al., 2017). The genus *Phaseolus* is thought to have undergone at least seven separate domestication processes. *P. vulgaris* and *P. lunatus* have had two independent and isolated domestication episodes, whereas *P. coccineus*, *P. dumosus*, and *P. acutifolius* have had single independent domestication occurrences (Bitocchi et al., 2017).

### Lentil (*Lens culinaris* Medik.)

Lentil, along with wheat and barley, is one of the world’s earliest crops, with evidence of its cultivation found at Neolithic archaeological sites (Ljuština and Mikić, 2010). However, neither archaeological nor genomic investigations have been able to pinpoint the exact location of lentil domestication. It is believed that lentils were domesticated around 11,000 BC in the Near East, in Franchthi cave in Greece and in Tel Mureybet in Syria dated 8500–7500 BC, in a region known as “the cradle of agriculture” (Sonnante et al., 2009). *Lens culinaris* subsp. *orientalis* (Boiss.) Ponert is believed to be the progenitor species of lentil. *L. culinaris* subsp. *orientalis* is widely distributed in Southwest Asia (SWA) and sporadically found in Central Asia and Cyprus (Zohary et al., 2012). However, as it is not possible to differentiate wild from cultivated small-seeded lentil, the state of domestication of these carbonized remains is unknown. It is not until the 5th millennium BC that lentil seeds larger than the wild are found, which were unequivocally domesticated. Archaeological evidences indicate that the wild lentils were gathered in several sites in SWA (Liber et al., 2021) and outside of SWA. Lentils were the most extensively cultivated crops prior to the invention of pottery (Liber et al., 2021). Domesticated lentils were one of the first crops to be introduced to Europe and Egypt (Sonnante et al., 2009). Around 5000 BC, lentils had expanded throughout Europe’s cold and damp regions, as well as to India’s Harappan civilization (Fuller and Harvey, 2006; Zohary et al., 2012). The crop appears in the archaeological record in India around 2500 BC (Cubero, 1981); perhaps, it reached India about 3,000 years ago. From the Bronze Age (approximately 3300 BC to 1200 BC) onward, lentil was considered an important companion to wheat and barley. Lentil was also carried to the New World in the post-Columbus era.

### Pigeon Pea (*Cajanus cajan* L. Millsp)

Pigeon pea is an important legume of the semiarid tropics, mainly grown in Asia, Africa, and the Caribbean region (Fuller et al., 2019). *Cajanus cajanifolius* (Haines) Maesen, the wild progenitor of the pigeon pea (*Cajanus cajan* L. Millsp), has been found in Eastern Peninsular India alongside a diversified collection of other *Cajanus* species. Archaeological evidence reveals that pigeon pea was first domesticated in Indus valley civilization, in Orissa about the middle of the 2nd millennium BC, more precisely nearby Gopalpur and Golbai towns, where *Cajanus cajanifolius*, the wild progenitor species of pigeon pea, is found in wild habitats (Fuller and Harvey, 2006). Wild pigeon pea species population is majorly found in the interface of the forest-edge areas and savanna plains of the Telangana, Chattisgarh, and Odisha states of India (Fuller et al., 2019). On the basis of the presence of the vast diversity of pigeon pea wild species populations in Western Ghats and the Malabar Coast of India, linguistic evidences, fossil records, and wide uses in daily cuisine, India is supported as the center of origin of pigeon pea by other researchers as well (Vavilov, 1951; van der Maesen, 1991).

## Domestication Syndrome

In the domestication process, for the adaptability of the crop plants, morphological and agronomical traits are genetically altered (Rauf et al., 2010). In the race of enhancing crop yield, similar sets of traits are selected for making an artificial selection in a wide range of cultivated species, which is the so-called domestication syndrome and results in convergent evolution of crop species (Bitocchi et al., 2017). The domestication syndrome, in other words, refers to the genetic and phenotypic changes that many food crops have undergone as a result of this process (Hammer, 1984; Harlan, 1992). Seed dormancy loss, enhanced pod and seed size, erect growth habit, reduced toxins, early and synchronized flowering, and reduced seed dispersal loss are some of the most prevalent domestication features, although their importance varies by crop (Meyer and Purugganan, 2013). Many of the domestication-related traits in food legumes are comparable to those in cereals (Fuller, 2007). Other characteristics of legumes, such as mineral content shifts and carotenoid losses, were also altered, maybe accidentally, as a result of adverse effects or selection for improved palatability (Fernández-Martin et al., 2014) and increases in tryptophan levels (Fernández-Martin et al., 2014). The importance of domesticated traits and the order in which they are chosen are expected to differ across food legumes and cereals. Domestication benefits include increased production, ease of harvest, and survivability in a range of environments.

Despite their domestication, domesticated crops have been subjected to selection for crop improvement traits (e.g., greater palatability and productivity) as well as varietal-specific feature diversification (e.g., fruit pigmentation variation, grain starch composition diversification, and adaptation to various climates and latitudes) (Olsen and Wendel, 2013). While studying the genetic basis of phenotypic changes during domestication, it is important to distinguish between the domestication attributes and additional improvement traits. The domestication traits are changes that occurred during the initial domestication process and are usually fixed within the crop species. Crop improvement characteristics, on the other hand, are often different among populations or cultivars of a crop (Olsen and Wendel, 2013). Although the distinction between domesticated traits and later improvement or diversification traits is not always evident, the latter traits are often discernible because they vary between varieties or landraces.

Domestication syndrome is obvious in several modifications from wild to domesticated plants, as it is in other legumes. In the dolichos bean, as in other grain legumes, fixed growth habit and photoperiod insensitivity are considered domestication syndrome features (Huyghe, 1998). Lablab, like other domesticated crops, exhibits a “founder effect” characterized by high phenotypic variability and low genetic variability, particularly in South Asian germplasm, whereas genetic diversity is higher in Africa (Maass et al., 2005; Maass et al., 2010; Venkatesha et al., 2013).

## Genetics of Domestication-Related Traits in Legumes

Research workers have uncovered the genes that govern some of the most critical morphological changes linked with domestication over the past decade. The method of finding these genes began with the identification of quantitative trait loci (QTL) in the segregating populations, followed by positional cloning and candidate gene analyses. Despite a modest number of well-recorded domestication genes, some commonalities are emerging. In Table 1, we have summarized the genes that have been linked to phenotypic alterations in features under selection during domestication. The domestication-related phenotypes were earlier thought to be influenced by recessive, loss-of-function alleles (Ladizinsky, 1985; Lester, 1989). However, QTL mapping studies and the cloning of a few domestication genes revealed rather an inconsistent pattern. Nonshattering appears to be a recessive trait in some cereals and food legumes (Takahashi and Hayashi, 1964; Harlan et al., 1973; Watanabe, 2005; Li et al., 2006b), and several QTL studies have suggested it to be nonrecessive (Doebley et al., 1990, 1994; Doebley and Stec, 1991; de Vicente and Tanksley, 1993; Burke et al., 2002; Li et al., 2006a; Wills and Burke, 2007).

**TABLE 1:** List of some of the prominent examples of domestication-related genes identified in food legume crops.

Crop	Genome size (Mbp)	Trait of interest	Gene/QTL involved	References
Chickpea (*Cicer arietinum*)	∼738	Early flowering	*efl-1*, *efl-2*, *efl-3*, *efl-4*	Nunavath et al.,^2020^
		Drought tolerance	*Dehydrin (DHN)*	Kumar et al.,^2020^
			QTL-Hotspot	Bharadwaj et al., 2021
		Plant development and abiotic stress tolerance	*CarLEA4*	Gu et al.,^2012^
		Abiotic stress	*CarERF116*	Deokar et al.,^2015^
		Biotic and abiotic stress tolerance	*Aquaporin* family	Deokar and Tar’an, 2016
		Plant growth habit	*Prostrate*	Aryamanesh et al.,^2010^
Soybean (*Glycine max*)	∼1085	Resistance to Asian soybean rust	*Rpp4C4 (PI459025B)*	Meyer et al.,^2009^
		Salt tolerance	*QTL qppsN.1*	Chen et al.,^2008^
Adzuki bean (*Vigna angularis*)	∼538	Waterlogging and biotic stresses	*ACT and ZMPP*	Chi et al.,^2016^
		Salinity–alkalinity and drought stresses	*Fbox*, *UBC*, and *PTB*	
Common bean (*Phaseolus vulgaris*)	∼588	Anthracnose resistance	Co-1 to Co-14	Choudhary et al.,^2018^
			Co-1, Co-x, Co-w	
			Co-3, Co-9, Co-y, Co-z, Co-10, and Co-15	
		Flowering time variation	*CLV2*	Raggi et al., 2019
		Resistance to bean common mosaic virus	*Gene I*	Melotto et al.,^1996^
Cowpea (*Vigna unguiculata*)	∼587	Stay-green	*Dro*	Muchero et al., 2013
		Fusarium wilt	*Fot3-1*	Pottorff et al., 2012
		Black seed coat	*MYB11*	Herniter et al., 2018
		Seed coat pattern and development	*Basic helix-loop-helix protein gene*, *WD repeat gene*, *E3 ubiquitin ligase gene*	Herniter et al.,^2019^
		Resistance to *Thrips tabaci* and *Franklin iellaschultzei*	*Thr-1*, *Thr-2*, *Thr-3*	Muchero et al., 2010
Peanut (*Arachis hypogaea*)	∼2,556	*Cercospora arachidicola* and *Aspergillus flavus* tolerance	*BjNPR1*, *Tfgd*	Sundaresha et al., 2016
		Salinity and drought stress	*AtHDG11*	Banavath et al.,^2018^
Lentil (*Lens culinaris*)	∼4,000	Boron tolerance	*MIP* gene (Lc09014)	Rodda et al.,^2018^
		*Ascochyta* blight resistance	*NBS-LRR*, *RLK*, *ral1* and *AbR1*	Sari et al., 2018
				Tar’an et al.,^2003^
		Anthracnose resistance	*LCt-2*	Eujayl et al.,^1999^
Mung bean (*Vigna radiata*)	∼540	*Bruchid* resistance	*VrPGIP, VrPGIP2*	Kaewwongwal et al., 2017
Pigeon pea (*Cajanus cajan*)	∼858	Determinacy	*CcTFL1*	Mir et al.,^2014^
		Heat response	*CcHsfA-1d* and *CcHsfA-2*	Maibam et al., 2015
		Stress tolerance	*WRKY family*	Singh et al.,^2019^
Faba bean (*Vicia faba*)	∼13,000	Light adaptation	*CHS and DOGT1 Oc1*, *Oc2*, *Oc3*	Yan et al.,^2019^
		Resistance to broomrape		Roman et al., 2002
Pea (*Pisum sativum*)	∼5,000	Drought tolerance	*DREB2A*	Jovanović et al., 2013
		Powdery mildew resistance	*Mlo*	Mohapatra et al., 2016
		Resistance to pea seed–borne mosaic virus	*Sbm-1*	Frew et al., 2002
		Resistance to *Fusarium* wilt	*Fw*	Dirlewanger et al.,^1994^
		Resistance to pea common mosaic virus	*Mo*	
White lupine (*Lupinus albus*)	∼924	Flowering time variation	*GI*, *FT*, and *SEP*	Rychel et al., 2019
Moth bean (*Vigna aconitifolia*)		Adzuki bean weevil (*Callosobruchus chinensis* L.) resistance	*Rcc*, *qVacBrc2.1*, *qVacBrc5.1*	Somta et al., 2018
Blue lupine (*Lupinus angustifolius*)	924	Resistance to *Diaporthetoxica*	*Phr1*	Yang et al., 2002
		Resistance to *Colletotrichum gloeosporiodes*	*Lanr1*	Yang et al., 2004
Grass pea (*Lathyrus sativus*)	8,200	Resistance to *Ascochyta* blight	*2 QTLs*	Skiba et al., 2004
Black gram (*Vigna mungo*)	574	Resistance to yellow mosaic virus	*VMYR1*	Basak et al.,^2004^

### Pod Shattering

Domestication features provide benefits, such as a higher yield, ease of harvest, and survival in a variety of conditions. However, these characteristics may reduce the fitness of the crop in the natural environment (Doebley et al., 2006). In wild legumes, pod shattering, for example, is a crucial mechanism for distributing seeds and supporting survival and reproduction. Shattering seeds allows seeds to disperse over longer distances, allowing them to settle in locations far from the original maternal plant diseases and siblings. The natural predisposition for seed dispersal, on the other hand, is an unfavorable trait in crops because it results in severe losses and causes inefficient harvesting (Vaughan et al., 2007).

In numerous cereal crops, particularly *Arabidopsis*, the transcriptional networks that promote shattering have been widely explored. However, we know very little about the molecular control of shattering behavior in legumes. Several legumes, including soybeans, have been studied for genetic control of pod shattering (Bailey et al., 1997; Liu et al., 2007; Funatsuki et al., 2008; Suzuki et al., 2009; Suzuki et al., 2010; Funatsuki et al., 2014; Hofhuis et al., 2016): common bean (Abd El-Moneim, 1993; Koinange et al., 1996), pea (Blixt, 1972; Weeden et al., 2002; Weeden, 2007), cowpea (Aliboh et al., 1996; Mohammed et al., 2009; Andargie et al., 2011; Kongjaimun et al., 2012; Suanum et al., 2016), lentil (Ladizinsky, 1979; Tahir and Muehlbauer, 1994; Fratini et al., 2007), narrow-leaf lupine (Tahir and Muehlbauer, 1994; Kongjaimun et al., 2012), adzuki bean (Isemura et al., 2007; Kaga et al., 2008), and common vetch (Abd El-Moneim, 1993; Dong et al., 2017).

In most of the legumes, the shattering of the pods has been found to be a dominant character controlled by one to two genes or QTLs. Several studies have shown that shattering resistance can be achieved by mutations in a single locus in narrow-leaf lupine (Boersma et al., 2009), soybean (Funatsuki et al., 2008), cowpea (Kongjaimun et al., 2012), and pea (Weeden, 2007). There are two genes that are involved in determining the recessive nonshattering feature in lupine (Nelson et al., 2006). The first gene, *lentus* (*le*), influences pod endocarp orientation, lowering the mechanical pressure necessary for pod shattering, whereas the second gene, *tardus* (*ta*), unites the dorsal and ventral pods, preventing them from being separated (Boersma et al., 2009).

Pod shattering in chickpea is reported to be controlled by one recessive gene (Kazan et al., 1993) or multiple loci (Ladizinsky, 1979). The genetic control of pod shattering in cowpea is rather not very clear. It is found to be controlled by a single gene, *Dhp*, or by a combination of dominant and recessive alleles of numerous genes (Aliboh et al., 1996). The loss of suture and pod wall fibers that is regulated by the *St* locus causes pod shattering in the common bean (*Phaseolus vulgaris*) (Koinange et al., 1996). In soybean, a significant QTL influencing pod shattering, *qPHD1*, was discovered (Bailey et al., 1997; Funatsuki et al., 2006; Liu et al., 2007; Funatsuki et al., 2008; Kang et al., 2009). Besides *qPHD1*, several other minor QTLs have been reported in soybean as pod-shattering regulators (Kang et al., 2009; Yamada et al., 2009; Hofhuis et al., 2016). *qPHD1* promotes pod dehiscence by modulating the cell-wall components in the inner sclerenchyma either by influencing the main structure of lignin or by changing lignin deposition patterns. A loss-of-function of this gene was discovered to confer pod-shattering resistance (Dong et al., 2014). An NAC gene *SHAT1-5*, which is similar to *A. thaliana*’s *NST1*, was discovered to give pod-breaking resistance in soybean. By stimulating the lignification of fiber cap cells in pod sutures, *SHAT1-5* regulates secondary cell-wall development (Dong et al., 2014).

The characteristic feature of wild peas is dehiscing pods, providing quick and distant seed dispersal (Zaytseva et al., 2017). It is hard for plants with nondehiscent pods to survive in the wild, and plants with dehiscing pods are hard to be harvested. In terms of pod dehiscence, this creates a condition of disruptive selection, compelling wild peas to stay wild, cultivated peas to stay cultivated, and products of uncommon crosses between them to join either of the two gene pools. Seed dormancy is another essential adaptation of wild peas to their insecure environments (Weeden, 2007).

### Seed Dormancy

Seed dormancy is one of the common traits of domestication syndrome across crops. A reduction in seed dormancy is connected with domestication (Meyer and Purugganan, 2013; Smýkal et al., 2014; Purugganan, 2019). Seed germination timing is crucial in the natural world as germination at the wrong period can result in reduced survival and fitness (Smýkal et al., 2014; Finch-Savage and Footitt, 2017). Seed dormancy is concerned with seed dispersion and the reduction of resource conflicts between mother and offspring, as well as environmental synchronization (Penfield, 2017). Extended seed dormancy, while beneficial in natural ecosystems, is not a desirable characteristic for crops (Smýkal et al., 2014; Purugganan, 2019). In cultivated legumes, seed dormancy reduces the pace of germination, resulting in uneven germination and, as a result, poorer yields (Ladizinsky, 1987; Abbo et al., 2008). There are two major types of seed dormancy, namely, physical seed dormancy (hardseededness) and physiological (chemical changes over the period) seed dormancy. Seed dormancy is controlled by environmental factors such as light, temperature, moisture, and duration after fruit ripening. The balance between gibberellic acid (GA) and abscisic acid (ABA) affects seed dormancy/germination. ABA maintains dormancy, and GA causes halt of dormancy and promotes germination (Dwivedi et al., 2021). Physical seed dormancy also has a negative impact on the seed’s ability to absorb water, which is critical in the processing of legume foods (Smýkal et al., 2014).

The underlying mechanisms for seed dormancy in pulses are not well studied yet, except a very few. Physical dormancy seems the most common source of dormancy in legumes, whereas physiological dormancy is a feature in several legume and nonlegume species (Martin, 1946). However, in comparison with other legumes, a loss of seed dormancy and reduction of pod shattering are not considered the main key domestication trait in chickpea. A single major QTL *pectin acetylesterase 8 (PAE-8-2)* controls seed dormancy in common bean, and a 5-bp frameshift mutation in pectin acetylesterase-8-2 is a putative causative variable underlying seed imbibition (Soltani et al., 2021). In *Vigna vexillate*, a major QTL, i.e., *qSdwa3.1*, positively affects seed water absorption (Amkul et al., 2020). *KNAT7-1*, a class II KNOX gene, is identified to affect seed dormancy in green gram (Laosatit et al., 2022). In green gram seeds, a high level of α-amylase activity is found positively associated with seed dormancy (Lamichaney et al., 2018).

### Growth Habit

Significant changes in plant architecture have happened during the domestication process. Determinacy is an important agronomic characteristic associated with food legume domestication. Determinate vegetation has an advantage over indeterminate vegetation because it devotes all assimilates to reproductive growth (Huyghe and Ney, 1997). Because of their suitability for mechanical harvesting, determinate cultivars are favored over indeterminates (Boote et al., 2003). Determinate growth habit can overcome the lodging problem in some legume crops (Duc et al., 2015). Indeterminate types are characterized by vegetative buds at terminal meristems and stem apices, which keep on growing in the length of the stem and flower and produce pods until temperature and humidity allows (Bradley et al., 1997; Tian et al., 2010). Semideterminate plants have similar development tendencies to indeterminate plants, but their terminal meristems are terminated by flower buds. In determinate kinds, the transition of terminal meristems from a vegetative to a reproductive state results in the production of a terminal flower, and as a result, vegetative growth stops blooming or only lasts for a brief time (Bernard, 1972; Bradley et al., 1997).

Determinate types are available in chickpea, soybean, cowpea, broad bean, common bean, and pigeon pea. Genetic control of stem growth habit has been studied in various legumes, including chickpea (Van Rheenen et al., 1994; Hegde, 2011; Harshavardhana et al., 2019; Ambika et al., 2021), soybean (Woodworth, 1933; Bernard, 1972; Thompson et al., 1997), pigeon pea (Waldia and Singh, 1987; Gupta and Kapoor, 1991; Dhanasekar et al., 2007), pea (Swiecicki, 1987), faba bean (Sjodin, 1971; Filippetti, 1986), lupine (Mikolajczyk et al., 1984), common bean (Singh, 1981), and mung bean (Khattak et al., 2004).

Determinacy was found to be a recessive trait in *Cicer arietinum* (Van Rheenen et al., 1994; Hegde, 2011; Harshavardhana et al., 2019; Ambika et al., 2021), *Glycine max* (Bernard, 1972), *Vicia faba* (Filippetti, 1986), and *Cajanus cajan* (Waldia and Singh, 1987; Gupta and Kapoor, 1991). The two nonallelic genes regulate stem growth habit, which is designated as *Dt1/dt1, Dt2/dt2* with *Dt1* epistatic to *Dt2* as well as *dt2* in chickpea (Hegde, 2011), soybean (Bernard, 1972), and pigeon pea (Waldia and Singh, 1987; Gupta and Kapoor, 1991). Additional studies on comparative genomics and *CcTFL1* expression profiling have shown that the gene *CcTFL1* is the candidate gene for determinacy in pigeon pea (Mir et al., 2014). In essence, *GmTFL1* in soybean, *PvTFL1* in common bean, and *CcTFL1* in pigeon pea were found to contain the same genomic region (Mir et al., 2014). It has been discovered that mutations in a homolog of the Arabidopsis *TFL1* gene create the determinate mutant (*det*) in pea (Foucher et al., 2003). A novel mutation in cowpea *TFL1* homolog (*VuTFL1*) determines the determinate growth habit. A nonsynonymous point mutation in exon 4 at position 1,176 resulted in transversion of cytosine (C) to adenine (A) which translated to the substitution of proline by histone (Pro-136 to His), and which resulted in to a determinate mutant of cowpea (Dhanasekar and Reddy, 2015).

The development of high yielding cultivars with a determined growth habit in a photoperiod insensitive background is one of the primary goals of breeding in grain legume crops. The genetics of photoperiod sensitivity and growth habit were studied in two crosses of dolichos bean, namely, HA 4 × GL 103 and HA 4 × GL 37, which were developed from parents contrasting for photoperiod sensitivity and growth habit. It was found that a monogenic biallelic locus controls photoperiodic response to flowering time, with photoperiod sensitivity dominating insensitivity (Keerthi et al., 2014).

### Flowering Time

Days-to-flowering is an important domestication feature that distinguishes the cultivated grain legumes from its wild relatives. For high grain yield and widespread cultivation, cultivated plants were domesticated to flower earlier than wild plants. Flowering time is controlled by a complex network that includes photoperiod, vernalization, gibberellin, autonomy, and the aging pathway (Amasino, 2010; Srikanth and Schmid, 2011).

In soybean, early flowering was preferred during domestication, as evidenced by the fact that cultivated soybeans flower earlier than wild relatives (Dong et al., 2001; Liu et al., 2007; Wen et al., 2009). Early flowering and maturity are conferred by the *E* series of maturity loci (*E1* to *E9*), especially under noninductive circumstances (Cober and Morrison, 2010). The early blooming alleles at the *E* loci, except the *E6* and *E9* loci, are recessive (Watanabe et al., 2012; Kong et al., 2014). *E2* is a putative floral repressor that encodes a GI homolog (Watanabe et al., 2011).

The recessive alleles of *early flowering 1* (*Efl1*) to *Efl4* produce early flowered chickpea (Gaur et al., 2015). The *Efl1* allele was identified in the early flowering genotype ICCV96029 (Kumar et al., 2000), which has been used as an important donor in the major chickpea breeding programs. A flowering time QTL containing a tandem array of *FTa* and *FTc* genes is found to control flowering time in a variety of temperate legumes, including lupine (Nelson et al., 2006), *L. japonicus* (Gondo et al., 2007), alfalfa (Robins et al., 2007), *M. truncatula* (Pierre et al., 2008), chickpea (Cobos et al., 2009; Aryamanesh et al., 2010), and faba bean (Cruz-Izquierdo et al., 2012).

In pea, recessive alleles at the *HIGH RESPONSE* (*HR*) locus cause short duration (SD) to early flowering and diminish but do not eliminate photoperiod response. On the other hand, recessive alleles at the *STERILE NODES* (*SN*) loci give total day-length insensitivity (Murfet, 1985). Alleles at the HR locus are significantly associated with the number of days to flowering, with an average difference of 15.43 days between two detected haplotypes (Vanhala et al., 2016).

The other locus, i.e., *LATE Blossoming* (LF), reduces the flowering period on both long and short days. It appeared to be a divergent homolog of *TFL1* (Foucher et al., 2003). The *LF* gene is found to be deleted or inactivated by a nonsense mutation in extraordinarily early accessions (Foucher et al., 2003). Late flowering expression was found to have a significant effect on the number of days to flowering when analyzed on its own but not when a high response to a photoperiod haplotype was added to the model. A high response to a photoperiod haplotype and GSO together explained most of the detected variation in DTF (49.6%) (Vanhala et al., 2016). The fourth locus is *EARLY* (*E*). The dominant alleles of *E* confer early onset of blooming in different genetic backgrounds (Weller et al., 2012).

In related *Pisum* species, multiflowering racemes have been observed. White (1917) described *Pisum elatius* as having 2–3 blooms per peduncle, whereas *Pisum arvense* had three or more flowers per peduncle (Gritton, 1980). The vast variation in the number of flowers per node in pea and other Fabaceae suggests that several processes may be involved in flower quantity regulation per peduncle (Gaur and Gour, 2002; Talukdar, 2013). The Indian genetic stock VRP–500 (INGR15009) has three flowers per peduncle at several flowering racemes (Sanwal et al., 2016). Likewise, the single plant selection “VRPM-901-5” from the cross “VL-8 PC-531” bears five blooms per peduncle at several flowering nodes (Devi et al., 2018). VRPM-501, VRPM-502, VRPM-503, VRPM-901-3, and VRPSeL-1 are examples of plants that produce three flowers per peduncle at multiple flowering nodes.

## Strategy to Accelerate Crop Domestication

**FIGURE 3:**
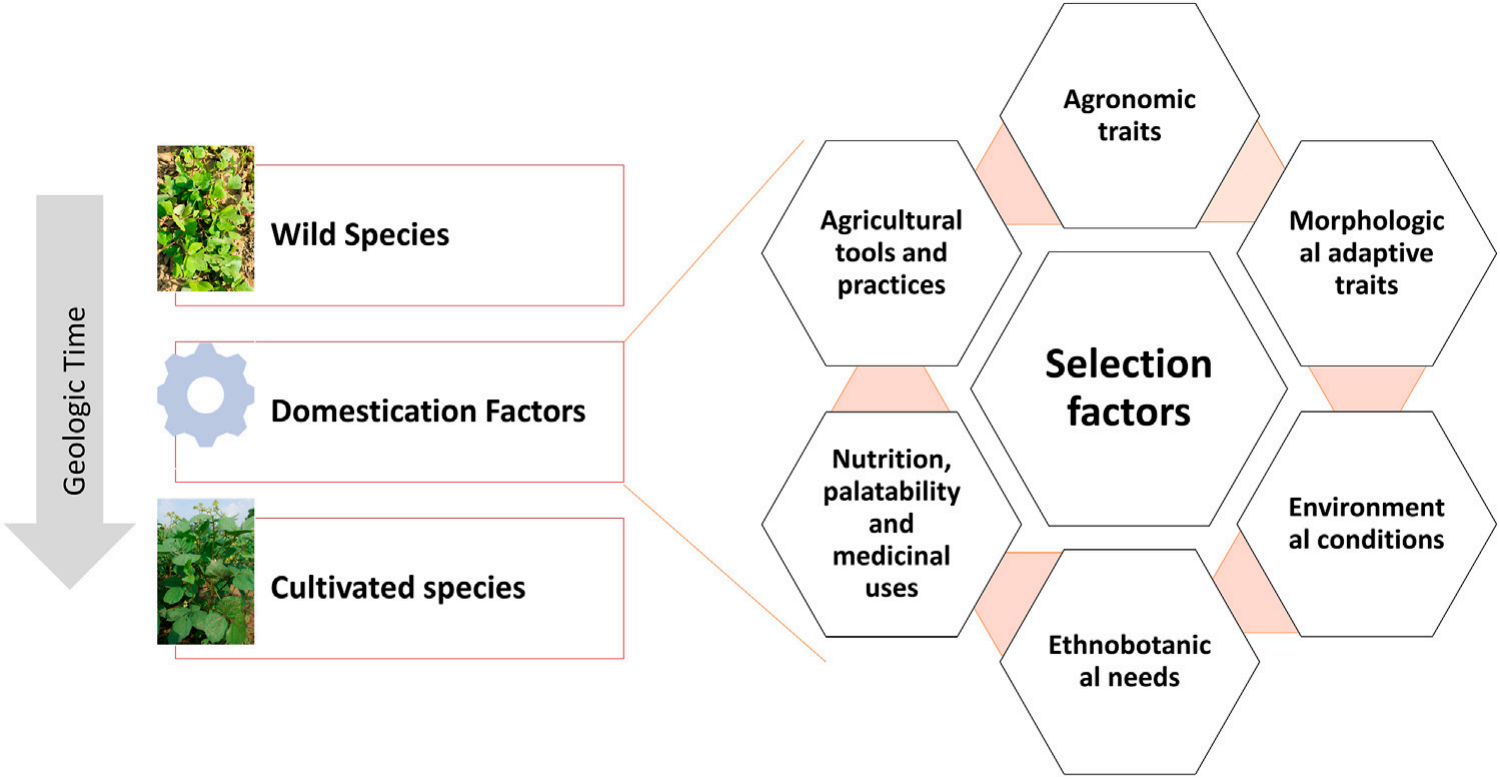
Illustration of the conventional domestication process and the selection factors involved.

**FIGURE 4:**
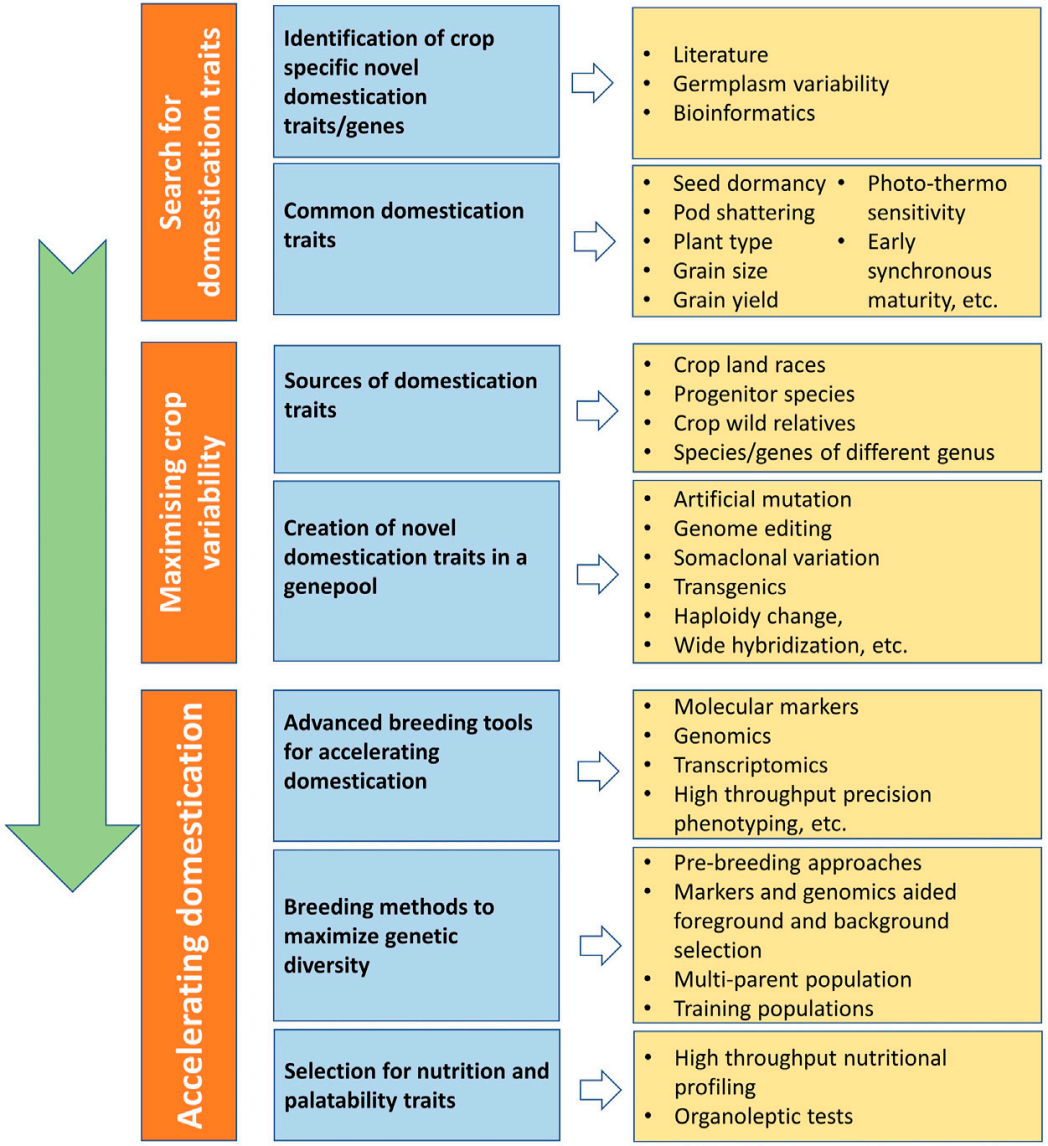
Scheme for accelerating the domestication process of new crops with maximizing genetic diversity.

Traditionally domesticated cultivars evolve through the interaction with multiple selection factors, artificial as well natural (Figure 3). Traits related to agronomic importance, nutrition, palatability, medicinal uses, agricultural tools and practices, and social and cultural values are majorly used for artificial selection. Plant morphological adaptive traits and resistance to biotic and abiotic stresses are the major natural selection pressures. Human preferences for crops and grain qualities keep changing because of social interactions, ongoing agronomic innovations, and environmental changes. Therefore, accelerated domestication of new food legume crops or further improvement of earlier domesticated or semidomesticated food legume crops is required to meet out the current needs amid fast changing demographic, edaphic, and climatic conditions. The Green Revolution is the recent example of accelerated domestication process particularly in rice and wheat and its impact on genetic diversity. As the Green Revolution only focused on a few genes (*Rht* genes of wheat and *sd1* of rice, it resulted in drastic diversity loss in the cultivated genepools of target crops and other crops as a side effect (Hedden, 2003). Uniformity in farmer fields enhanced dramatically because of the replacement of traditional cultivars by modern high yielding varieties, but this also turned crops more susceptible for biotic and abiotic stresses. It is well understood that the process of domestication has also caused allelic loss particularly for quantitative traits, which results in poor yield potential, quality, and adaptation (Van Tassel et al., 2020). Therefore, along with accelerating the domestication process, genetic base broadening has now become more important. A strategy for accelerating the domestication process and for enhancing genetic diversity in the new domesticates to meet the current requirements is highlighted in Figure 4.

Genome reshuffling, mutation, and the myriad of natural and artificial selection pressures keeps genome highly dynamic. However, selection and advancement of a fit genotype to the next generation and then establishing itself in a population are very slow processes. Recent understanding about crop population dynamism, recent technological advancements, and artificial intelligence has made the domestication process easier and faster. Recent developments in genomics, proteomics, transcriptomics, metabolomics, and phenomics have made much easier to identify domestication-related genes, to pinpoint in the genome, and to use them in marker-assisted introgression. Marker-assisted selection for foreground and background selection makes gene introgression easier while retaining and maximizing the background genetic diversity. High-throughput precision phenotyping in association with genomic selection can help in rapid genetic improvement (Van Tassel et al., 2022).

Domestication of crops and plant breeding led to the development of crops with a high yield, which is adjusted well to native growing circumstances. The idea of *de novo* domestication strategy includes the detection and introgression of genes or mutants essential for domestication success and acclimatization of newly developed cultivars (Khan et al., 2019). However, the domestication process resulted in some undesirable consequences such loss of genetic diversity, imbalance in nutritional status, and reduction in the taste of current food-producing crops. It is therefore essential to facilitate a sustainable agriculture system for accelerating crop genetic diversity and improving global food production. Some of the major factors involved in the *de novo* domestication process are discussed below.

### Crop Genebanks

Crop genebanks harbor a good amount of *ex situ* conserved genetic diversity in the form of landraces and wild species, which is merely utilized. Over one million samples of *ex situ* collections of grain legumes are conserved in various seed genebanks across the globe. This could serve as the firsthand source of the novel traits and alleles to be used in the development and improvement of cultivars. The centers of the crop origin and primary or secondary centers of diversity are considered the hotspot for genetic diversity as agroclimatic conditions favor the rapid evolution of new alleles. Therefore, such areas should be the target to search the novel traits in wild species, crop wild relatives, or landraces. Wild relatives of modern cultivated crops are considered an important source of novel alleles. Those traits that are not present in the entire cultivated genepool, the progenitor species or wild relatives, can be introduced from an alien source (other genera or crops) using nonconventional approaches such as random or site-specific mutagenesis.

### Genome Resequencing

For crop species with well-characterized reference genomes, genome-wide screening for selection signatures offers a potentially powerful complementary approach to the genetic mapping strategies. Advances in sequencing technologies and the reduction of their costs have supported the publication of numerous high-quality studies on crop domestication using genome resequencing. Varshney et al. (2019) documented fourfold reduction in diversity from wild genotypes to landraces, highlighting the loss of about 80% of genetic diversity by whole genome resequencing of 429 chickpea genotypes, identified allele(s) and genomic region(s) affected during domestication and postdomestication diversification, and identified 122 candidate domestication regions and 204 genes that underwent selection.

Whole genome resequencing of 302 wild, landrace, and improved soybean accessions detected a total of 121 domestication-selective sweeps, 109 improvement-selective sweeps, and 162 selected copy number variants of domestication-related traits (Zhou et al., 2015). From whole genome resequencing of 292 *Cajanus* accessions comprising of breeding lines, landraces, and wild species, selective sweeps responsible for reduction in genetic diversity under domestication and breeding were identified. From the comparison of wild species accessions with landraces and landraces with breeding lines, a total of 2,945 and 1,323 genomic regions, respectively, were identified with higher ROD values. The resequencing information also helped in identification of 666 and 1,643 genomic regions with low genetic variation consistent with positive selection during domestication and breeding, respectively (Varshney et al., 2017).

### Marker-Trait Association for Identification of Target Genomic Regions

Marker-trait association has played a significant role in accelerating crop domestication through trait identification and introgression in target genotypes. Conventional linkage mapping based on biparental mapping population and now genome-wide association studies (GWAS) and linkage disequilibrium (LD) mapping are the two major strategies currently being used by researcher for identifying statistically significant associations between phenotypes and genotypes or between domesticated traits and sequence variants.

Marker-trait association analysis provides the genetic basis for phenotypic variation, including gene locations, numbers, and magnitudes, and their mechanisms in a biparental segregating population. There has been substantial progress in mapping the QTLs/genes underlying crop domestication using these methodologies. This has enabled successful identification and cloning of genes underlying domestication traits. It was the first and perhaps the most widely used method for localizing the genetic basis of a trait. Lu et al. (2020) resequenced 424 soybean accessions, analyzed time of flowering and maturity through GWAS, and identified three significant association loci (*p* < 10^−8^) on chromosomes 11 and 12, subsequently referred to as Time of Flowering 11 (*Tof11*) and *Tof12*. Compared to wild soybean, soybean with loss of *Tof11* and *Tof12* function significantly reduces photoperiod sensitivity and significantly shortens the time to flowering and maturity under LD conditions. Furthermore, it is evident that, a change in phenology related to loss of *Tof12* function had a significant role in adaptation of wild soybean during a phase of initial cultivation and domestication. Lo et al. (2018) evaluated nine domestication-related traits (pod shattering, peduncle length, flower color, days-to-flowering, 100-seed weight, pod length, leaf length, leaf width, and seed number per pod). A high-density genetic map containing 17,739 single nucleotide polymorphisms was constructed and used to identify 16 QTL for these nine traits. On the basis of the annotations of the cowpea reference genome, genes within these regions were reported. Four regions with clusters of QTLs were identified, including one on chromosome 8 related to increased organ size. Swarm et al. (2019) conducted QTL mapping on domestication-related traits (DRTs) using 661 RILs from two populations with 5,000 polymorphic SNP markers in soybean. A total of 132 QTLs were detected, of which 51 were associated with selective sweeps previously related to soybean domestication. They identified 41 novel QTLs not detected in previous studies using smaller populations while also confirming the quantitative nature for several of the important DRTs in soybeans.

### Mutagenesis

Mutations are the ultimate source of variation. Mutagens are used to create random mutations, and specific mutations in the desired domestication gene can subsequently be identified. Mutants serve as means for identifying genes that control developmental decisions in plants like flowering, and crops with improved traits are being developed by screening for mutations induced in candidate genes. Dhanasekar and Reddy (2015) isolated and characterized a novel mutation in cowpea *TFL1* homolog (*VuTFL1*) affecting determinacy using gamma rays. Analyses of sequence variation exposed a novel SNP distinguishing the determinate mutants from the indeterminate types. The nonsynonymous point mutation in exon 4 at position 1,176 resulted from the transversion of cytosine (C) to adenine (A), leading to an amino acid change (Pro-136 to His) in determinate mutants. Using random mutagenesis, the *Btr1* gene in a wild barley accession was mutated, and this resulted in plants that resembled domesticated barley, with a nonbrittle rachis (Pourkheirandish, 2015). Thus, a loss-of-function mutation in a single domestication gene can indeed result in a domestication phenotype.

### Genome Editing for Domestication of Crops

In spite of their importance in ensuring food security, legumes are cultivated less owing to their several undesirable traits such as pod shattering, late flowering, and indeterminate growth habit (Zhang Y. et al., 2020). Although conventional breeding for quality enhancement of food legumes is challenging, genetic modification through guided nucleases is an ideal platform. This robust domestication is proposed not only to cope with the changing climate scenario but also to ensure food security to fellow citizens. The novel CRISPR/Cas-based gene editing is a powerful, precise, economic, efficient, multiplexed method to accelerate the domestication of food legumes. Targeting genes for accelerated domestication by genome editing involves reducing or abolishing gene function based on existing knowledge of the molecular function of the target gene. This requires that the genome of the target plant is sequenced to identify genes that are orthologs of known genes controlling domestication traits in related plants. CRISPR/Cas9 and TALEN could help in the development of novel traits through loss/gain of function of genes already available in the genome (Bedell et al., 2012). Of late, more advanced genome-editing tools having high precision with minimal unwanted genome modification, such as Base editors and Primer editors, are now available for genome modification (Anzalone et al., 2020). For many of the food legume crops, gene-editing methods are established because of their recalcitrance behavior at various phases of genome editing such as *in vitro* gene transfer and regeneration. For crops such as soybean, cowpea, and chickpea, gene-editing protocols are well established (Bhowmik et al., 2021). However, the utilization of this powerful technology has just started in the crop improvement, and results are yet to come.

CRISPR/Cas9 can precisely edit genes to improve genotypes and aid in accelerating the domestication process of new crops (Van Tassel et al., 2020). CRISPR-Cas can fine-tune and knock out master switches in undomesticated wild crops, enhance genomic diversity, and facilitate *de novo* domestication in one generation or a few generations. CRISPR/Cas9 is successfully utilized in soybean to develop a mutant of *GmFT2a* with delayed flowering time and with enhanced pod yield in the crop (Rasheed et al., 2022). The technology has been successfully utilized in trait improvement of few other nonlegume crops as well. This technology was used to domesticate the wild tomato (*Solanum pimpinellifolium* L.) by improving several critical agronomic and nutritional quality attributes. Five sets of genes, viz., *SP*, *SP5G*, *CLV3*, *WUS*, and *GP1*, were altered through the CRISPR-Cas9 modular cloning approach, resulting in a compact plant with early flowering, day-length neutral, enhanced fruit size, and high vitamin C levels (Li et al., 2018). The CRISPR/Cas9 system has been successfully used as an efficient tool for genome editing in *Oryza sativa*. It was used to mutate the *Gn1a*, *DEP1*, and *GS3* genes of rice, which have been reported to function as regulators of grain number, panicle architecture, and grain size, respectively. The T_2_ generation of the *gn1a*, *dep1*, and *gs3* mutants featured enhanced grain number, dense erect panicles, and larger grain size, respectively. Furthermore, semidwarf and grain with long awn phenotypes were observed in *dep1* and *gs3* mutants, respectively (Li et al., 2016). This has offered the potential to improve domestication traits.

Multiplex gene editing has shown promise in creating desired *de novo* domesticated tomato plant. Editing of six genes, viz., *SP* (*Self Pruning*), *Fw2.2* (*Fruit Weight 2.2*), *Ovate*, *Multiflora* (*MULT*), *Fas*, and *Lycopene Beta Cyclase* (*CycB*), resulted in a smart crop with improved fruit size, yield, and nutritional quality (lycopene contents) (Zsögönet al., 2018). Likewise, *Solanum pruinosa* (ground cherry), an orphan crop, was improved through the CRISPR-based technique involving several targeted genes (*SP*, *SP5G*, and *CLV1*), resulting in improved domestication characteristics (Lemmon et al., 2018). CRISPR gene editing can be used to develop desirable features in any crop after discovering genes that regulate domestication. The genome-editing techniques promise to be a useful tool in the plant breeding toolbox for domesticating new crops or trait improvement. The technology can dramatically accelerate the process of domestication.

### Reverse Genetics and Other Tools

There are several other methods and tools to generate novel traits and variability, such as TILLING (McCallum et al., 2000), somaclonal variation (Larkin and Scowcroft, 1981), hybridization (Seehausen, 2004), directed evolution (Currin et al., 2021), and alien gene transfer through transgenic approaches. RNA interference (RNAi) generally does not alter the gene, but it is used to silence or lowers the target gene expression, e.g., lowering the expression of the *LABA1* gene made the awns shorter and smaller to resemble the domesticated phenotype in rice (Hua et al., 2015).

To further utilize and find suitable traits and genotypes in the artificially generated diversity, advanced scientific tools can be explored. Molecular markers and genomics-assisted selection offers a great help in rapid identification superior genotypes. Though artificially generated genetic diversity or genotypes with novel traits are finally tested in natural target environments, transcriptomics, proteomics, and metabolomics can help in the selection and functional validation of selected genotypes. The artificial intelligence and statistical analysis tools further help in handling larger datasets and decision-making.

## Conclusion

Domestication and evolution of wild ancestors into today’s elite cultivars are important contributors to global food supply and agricultural crop improvement. During domestication and evolution, many crop species underwent significant morphological and physiological modifications. According to genetic studies, a few genes control DRTs, and these genes frequently have a major impact on plant phenotype. The identification and knowledge of loci that are responsible for significant alterations that occur as a result of domestication might aid the quick domestication of novel crops. Shattering resistance, seed dormancy loss, stem growth behavior, and a shorter flowering period are the key domestication characteristics of food legume crops. Identification of genes involved in these functions, as well as the explanation of the molecular pathways involved in these processes, is required to gain deeper understanding of these fundamental characteristics. Apart from accelerating domestication, it is realized that enhancing the crop genetic diversity in farm landscapes is now more important to sustain the crop yield amid changing climatic conditions and diverse human needs. Recent advances in science and technology offer a great help in the identification and functional annotation of genes having a great impact on domestication. Technologies also help in the rapid enhancement of genetic diversity in crop genepools to keep crops more adaptive to the changing environmental conditions and human need.

## Footnotes

1 Soybeans (Glycine max)-The Plant History of the Marvelous Soybean. Available online: https://www.thoughtco.com/plant-history-of-the-soybean-3879343 (Accessed 12 April 2022).

2 Pea (Pisum sativum L.) Domestication-the history of peas and humans. Available online: http://archaeology.about.com/od/Domesticated-Plants/fl/Pea-Pisum-sativum-L-domestication the-history-of-peas-and-humans.htm (Accessed 28 April 2022).
